# MEF2D Functions as a Tumor Suppressor in Breast Cancer

**DOI:** 10.3390/ijms25105207

**Published:** 2024-05-10

**Authors:** Xiaoxia Wang, He Shen, Yanmin Chen, Yali Zhang, Jianmin Wang, Song Liu, Bo Xu, Hai Wang, Costa Frangou, Jianmin Zhang

**Affiliations:** 1 Department of Cancer Genetics and Genomics, Roswell Park Comprehensive Cancer Center, 665 Elm Street, Buffalo, NY 14203, USA; xiaoxia.wang@roswellpark.org (X.W.); he.shen@roswellpark.org (H.S.); yanmin.chen@roswellpark.org (Y.C.); 2 Department of Biostatistics and Bioinformatics, Roswell Park Comprehensive Cancer Center, 665 Elm Street, Buffalo, NY 14203, USA; yali.zhang@roswellpark.org (Y.Z.); jianmin.wang@roswellpark.org (J.W.); song.liu@roswellpark.org (S.L.); 3 Department of Pathology, Roswell Park Comprehensive Cancer Center, 665 Elm Street, Buffalo, NY 14203, USA; bo.xu@roswellpark.org; 4 Department of Molecular and Cellular Biology, Roswell Park Comprehensive Cancer Center, 665 Elm Street, Buffalo, NY 14203, USA; hai.wang@roswellpark.org

**Keywords:** MEF2D, breast cancer, tumor suppressor, cellular transformation, RNA-seq

## Abstract

The myocyte enhancer factor 2 (MEF2) gene family play fundamental roles in the genetic programs that control cell differentiation, morphogenesis, proliferation, and survival in a wide range of cell types. More recently, these genes have also been implicated as drivers of carcinogenesis, by acting as oncogenes or tumor suppressors depending on the biological context. Nonetheless, the molecular programs they regulate and their roles in tumor development and progression remain incompletely understood. The present study evaluated whether the MEF2D transcription factor functions as a tumor suppressor in breast cancer. The knockout of the MEF2D gene in mouse mammary epithelial cells resulted in phenotypic changes characteristic of neoplastic transformation. These changes included enhanced cell proliferation, a loss of contact inhibition, and anchorage-independent growth in soft agar, as well as the capacity for tumor development in mice. Mechanistically, the knockout of MEF2D induced the epithelial-to-mesenchymal transition (EMT) and activated several oncogenic signaling pathways, including AKT, ERK, and Hippo-YAP. Correspondingly, a reduced expression of MEF2D was observed in human triple-negative breast cancer cell lines, and a low MEF2D expression in tissue samples was found to be correlated with a worse overall survival and relapse-free survival in breast cancer patients. MEF2D may, thus, be a putative tumor suppressor, acting through selective gene regulatory programs that have clinical and therapeutic significance.

## 1. Introduction

Breast cancer is a common and heterogeneous disease with several subtypes associated with different clinical behaviors [1]. While the survival rates of patients diagnosed with breast cancer have progressively increased over time as a result of improvements in early tumor detection and treatment, breast cancer remains the leading cause of cancer-related death among women worldwide [2]. Breast cancer arises through a multi-step, mutagenic process whereby mammary epithelial cells (MECs) acquire a common set of properties, including unlimited proliferation potential, self-sufficiency in growth signals, resistance to apoptosis, and the ability to evade the immune system [3]. Although these “hallmarks of cancer” provide an organizational framework for understanding breast cancer and other neoplastic diseases in terms of a common set of underlying cellular parameters, the causal driver genes responsible for the transformation of cell populations into malignant-like cells and tumor cells are often unknown. Therefore, a better understanding of the cellular and molecular mechanisms associated with the initiation and progression of breast cancer is a prerequisite for developing new diagnostic and prognostic biomarkers, as well as targeted therapeutic strategies.

Cancer driver genes are broadly classified into oncogenes and tumor suppressor genes (TSGs) [3]. Oncogenes typically harbor gain-of-function mutations that activate the protein, leading to uncontrolled cell growth or proliferation. Conversely, TSGs play a crucial role in restraining inappropriate cell growth and division. In cancer, there is a strong positive selection pressure for deactivating somatic mutations. However, some driver genes can exhibit both tumor-suppressor and oncogene properties, depending on the context. While it is generally accepted that highly recurrent somatic alterations signify changes that are important for tumor development, the causal perturbations underlying tumor genesis are often confounded by the extensive size of alterations and the large number that are incidental to the tumor phenotypes. Furthermore, in many instances, driver genes are epigenetically regulated rather than being a result of mutational events, thus, they would not be identified by conventional genome sequencing methods.

The dysregulation of or mutations involving transcription factors have long been recognized in the development of breast cancer. Alterations in these key regulatory molecules can result in aberrant gene expression that leads to the blockade of normal cellular differentiation and cell death gene expression programs. For example, several transcription factors have been identified as drivers of breast cancer, including ARNT2, COX7RP, EGR3, FOXA1, FOXC2, FOXM1, FOXO3, NR3C2, and ZNF652. These transcription factors are involved in various cellular processes, including cell division, senescence, cell cycle regulation, and immune cell infiltration in breast cancer [4,5,6,7].

Myocyte enhancer factor 2D (MEF2D) belongs to the MADS-box family of transcription factors. In mammals, the MEF2 family of transcription factors comprises four genes: MEF2A, MEF2B, MEF2C, and MEF2D. These genes exhibit variations in their temporal and tissue-specific expression patterns, but are co-expressed throughout developing and adult tissues, such as skeletal muscle [8,9]. MEF2 proteins exhibit significant amino acid similarity within their DNA-binding domains and can bind to similar cis-acting sequences, thereby obscuring the mechanisms by which these factors control specific target genes. Nonetheless, the lack of conservation in the carboxyl-terminal transactivation domain among the four distinct MEF2 isoforms, coupled with the diverse phenotypes observed in different MEF2-deficient vertebrate models, implies that each MEF2 isoform independently controls specific gene programs.

The MEF2 proteins participate in diverse physiological processes, including neural differentiation, cardiac morphogenesis, blood vessel formation, and the maintenance of several vertebrate tissue types [10,11,12,13,14]. Notably, an increasing number of studies have detected the overexpression of MEF2D in non-small cell lung cancer (NSCLC) [15], colorectal cancer [16], hepatocellular carcinoma [17], osteosarcoma [18], and glioma [15]. Correspondingly, high levels of MEF2D expression have been shown to be correlated with a poor patient prognosis, and MEF2D suppression has been shown to decrease the proliferation of hepatocellular carcinoma [17], osteosarcoma [18], and glioma [19]. In contrast, MEF2D was recently identified as a TSG in rhabdomyosarcoma [20] and low-grade uterine leiomyosarcomas [21], thus suggesting that MEF2D plays a cell-type-specific role in cancer cells. In the context of breast cancer, decreases in MEF2D mRNA levels have been observed in tumor samples [22], which is suggestive of a potential role of MEF2D as a tumor suppressor. The extent to which MEF2D inhibits the conversion of mammary epithelial cells into neoplastic cells and its functional significance in the initiation and progression of breast cancer are poorly understood.

In the present study, we investigated the role of MEF2D in mammary epithelial cell (MEC) transformation and tumorigenesis and provided mechanistic insights into the dysregulation of MEF2D in breast cancer cells. Most human cancer cell lines currently available do not accurately replicate the genetic, molecular, and phenotypic changes observed in tumor cells from individual patients. Therefore, we used non-transformed murine mammary epithelial EpH4 and HC11 cells to explore the transformation activity of MEF2D and to identify the functionally related molecular characteristics associated with different cellular phenotypes. Using these model systems, we found that MEF2D contributes to cell proliferation by modulating the loss of contact inhibition and progression of the cell cycle, and by facilitating cell migration and inducing the formation of abnormal structures in three-dimensional (3D) cultures. Mechanistically, we found that a loss of MEF2D led to aberrant activation of the AKT/ERK signaling pathway, resulting in the induction of the epithelial-to-mesenchymal transition (EMT). MEF2D also led to the disruption of contact inhibition due to the abnormal activation of YAP. Consistent with these findings, the downregulation of MEF2D in human breast cancer samples and cell lines was found to be correlated with tumor aggressiveness, highlighting the therapeutic significance of our findings.

## 2. Results

### 2.1. Loss of MEF2D Expression Promoted MEC Proliferation and Migration

To investigate the role of MEF2D in the transformation of MECs, we used the CRISPR-Cas9 system to create MEF2D knockout cell lines in the EpH4 and HC11 murine mammary epithelial cells. The EpH4 cell line was derived from spontaneously immortalized mouse mammary gland epithelial cells isolated from a BALB/c mouse. EpH4 cells can fully differentiate into spheroids when cultured under 3D growth conditions [23]. Similarly, the HC11 mouse breast epithelial cell line undergoes differentiation when stimulated with lactogenic hormones [23]. Unlike breast cancer cell lines, the EpH4 and HC11 models are ideal for studying signaling networks that control differentiation and transformation within the same cell system.

**Figure 1:**
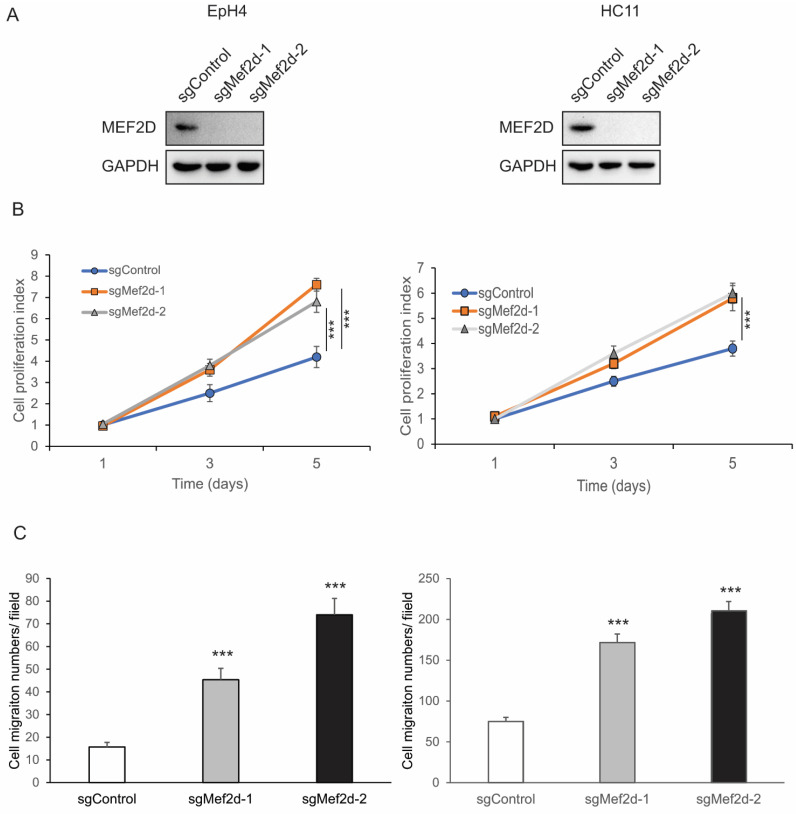
**Knockdown of MEF2D-increased cell proliferation and migration in murine EpH4 and HC11 cells.** (**A**) Immunoblots demonstrating the knockdown efficiency of MEF2D in EpH4 and HC11 cells. GAPDH was used as a loading control. (**B**) Data for cell proliferation assays performed at day 1, 3, and 5 in sgControl- or sgMEF2D-transduced EpH4 and HC11 cells. *** *p* < 0.001. (**C**) Quantification of results for Boyden chamber cell migration assays of sgControl or sgMEF2D EpH4 and HC11 cells. *** *p* < 0.001.

Two different experimentally validated sgRNAs targeting MEF2D were used to transduce the Cas9-expressing EpH4 and HC11 cell lines, and an immunoblot analysis confirmed the absence of MEF2D protein levels in the MEF2D knockout cells (sgMEF2D) in their comparisons to control cells (Figure 1A). The cell proliferation assay results revealed that loss of MEF2D increased cell proliferation in the EpH4 and HC11 cells compared with the control cells (Figure 1B). In addition, the perturbation of MEF2D increased cell migration (Figure 1C; Supplementary Figure S1A,B). These findings indicate that the loss of MEF2D function promotes epithelial cell proliferation and migration.

### 2.2. MEF2D Knockout Induced EMT and Activation of the AKT/ERK Pathway

**Figure 2:**
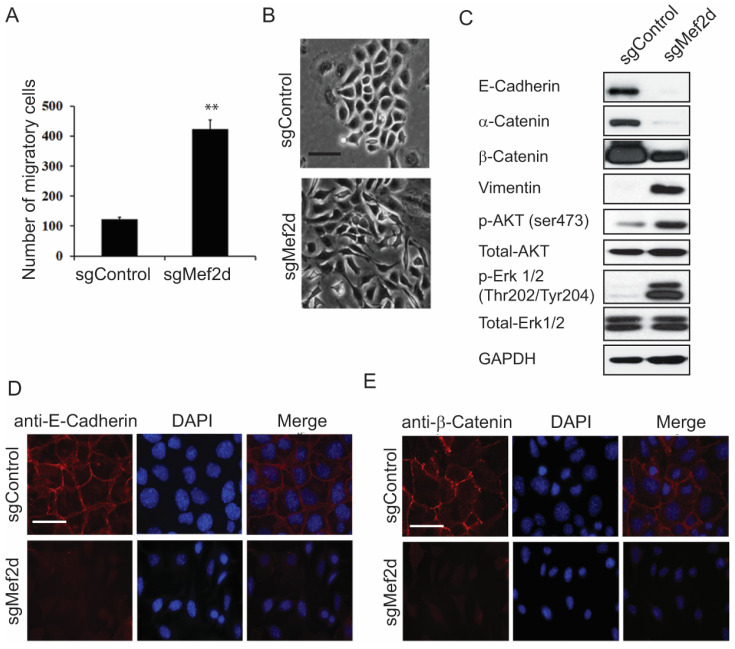
**Loss of MEF2D induced EMT.** (**A**) Quantification of wound healing assays demonstrated that loss of MEF2D increased EpH4 cell migration. ** *p* < 0.01. (**B**) Morphological changes showed MEF2D knockdown induced a typical epithelial to fibroblast-like mesenchymal phenotype on monolayer cultures. Scale bars = 200 µm. (**C**) MEF2D depletion resulted in loss of epithelial markers (E-cadherin, α-catenin, and β-catenin) and mesenchymal markers (vimentin), as well as activation of AKT/ERK. GAPDH was used as a loading control. The membrane-localized E-cadherin (**D**) and β-catenin (**E**) in sgMEF2D cells were down-regulated compared with control cells, as revealed by immunofluorescence microscopy. Scale bars = 50 µm.

To further investigate the role of MEF2D in MEC mobility, we performed a wound healing assay. As shown in Figure 2A and Supplementary Figure S1C, the number of migrated cells significantly increased in the sgMEF2D EpH4 cells compared with the control cells, suggesting that the loss of MEF2D markedly enhanced cell migration. Notably, MEF2D knockout induced morphological alterations in the EpH4 cells, including cell elongation and scattering, resulting in a fibroblast-like mesenchymal phenotype. In contrast, the control cells maintained a characteristic polarized epithelial morphology (Figure 2B), suggesting that MEF2D knockout induced the epithelial cells to undergo the EMT.

The overexpression of MEF2D has been shown to promote invasion and the EMT in colorectal cancer cells [16]. The EMT is a process that contributes to breast cancer progression and metastasis and involves the conversion of epithelial cells into mesenchymal cells, which exhibit increased invasiveness and migratory capabilities [24]. However, the EMT process is complex and involves the coordinated expression of multiple transcription factors, such as SNAI2, ZEB1/2, TCF4, and TWIST1/2. Accordingly, to investigate whether MEF2D modulates the EMT, we initially evaluated the protein expression levels of the epithelial and mesenchymal markers. Consistent with the observed cell morphological changes, a Western blot analysis revealed that MEF2D knockout resulted in dramatic reductions in the E-cadherin and α- and β-catenin epithelial markers, and gains in the vimentin mesenchymal marker were observed (Figure 2C). In addition, compared with the control cells, there were increases in the levels of p-AKT and p-ERK in the sgMEF2D cells, without obvious changes in the total expression of AKT and ERK (Figure 2C), suggesting that the loss of MEF2D induced the EMT, possibly through the activation of the AKT/ERK pathway in the EpH4 cells. Consistent with these findings, immunofluorescence staining revealed that MEF2D depletion resulted in the downregulation of E-cadherin and β-catenin expression on the cell membrane (Figure 2D,E).

### 2.3. Depletion of MEF2D Induced Epithelial Cellular Transformation and Tumor Growth

**Figure 3:**
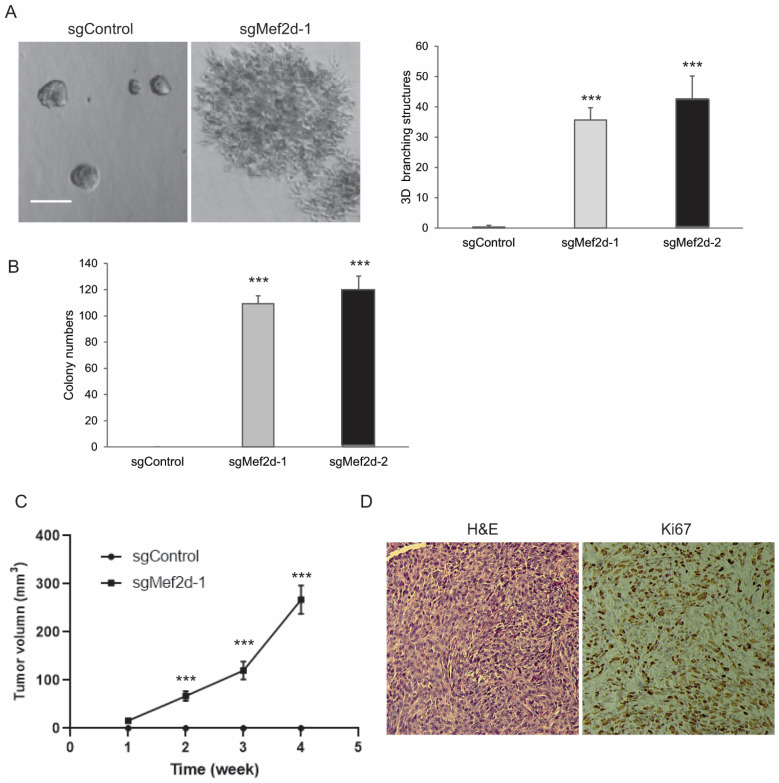
**Knockdown of MEF2D induced anchorage-independent growth of EpH4 cells in soft agar and tumor formation in vivo.** (**A**) Representative images and quantification of 3D acini formation of sgControl or sgMEF2D EpH4 cells. Scale bars = 200 µm. *** *p* < 0.001. (**B**) Quantification of colony formation in soft agar of sgControl or sgMEF2D EpH4 cells. Scale bars = 100 µm. *** *p* < 0.001. (**C**) sgControl or sgMEF2D cells were injected into 6–8-week-old SCID mice (n = 6). Tumor volumes were measured every 7 days. sgControl cells did not form tumors. *** *p* < 0.001. (**D**) Representative images of H&E and Ki67 immunohistochemistry (IHC) staining for sgMEF2D EpH4 cell-generated tumors (×200).

Three-dimensional basement membrane cultures have been widely used to model the architecture of the epithelium in vitro [25]. To determine the effects of the loss of MEF2D function on the epithelium architecture, we performed a 3D morphogenesis assay of the EpH4 cells grown in Matrigel. Compared to the sgControl sphere-like acini formation, MEF2D knockdown promoted the development of enlarged and branching 3D structures (Figure 3A). As expected, the EpH4 control cells failed to form anchorage-independent colonies in the soft agar. However, the loss of MEF2D led to a strong capacity for colony formation in the soft agar, which is a hallmark of transformation (Figure 3B; Supplementary Figure S2A).

Next, to assess the effects of MEF2D on in vivo mammary-tumor-forming potential, sgMEF2D or sgControl cells were injected into the mammary fat pads of SCID mice. Consistent with previous studies [26], the EpH4 control cells did not form tumors over the course of the experiment (Figure 3C). In contrast, the sgMEF2D cells generated palpable tumors within two weeks of injection, and the tumors grew at a high proliferative rate in vivo (Figure 3C). As shown in Figure 3D, the loss of MEF2D function induced a high-grade mammary tumor and malignant spindle cell proliferation. Taken together, these findings suggest that MEF2D is a TSG in breast cancer and that the loss of MEF2D induces cell transformation in vitro and tumor development in mice.

### 2.4. MEF2D Knockdown Led to Loss of Contact Inhibition through YAP Activation

**Figure 4:**
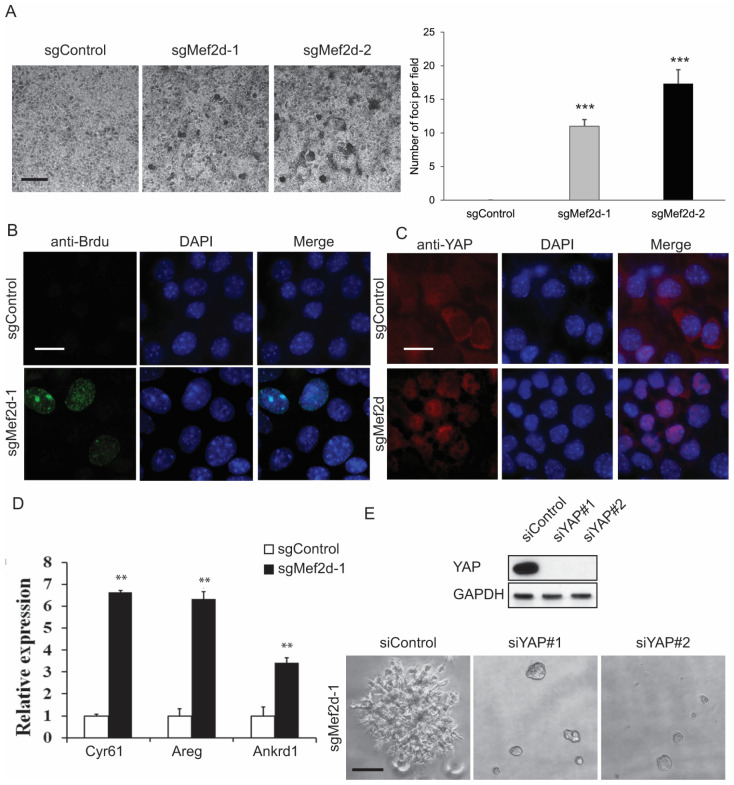
**MEF2D depletion promoted loss of contact inhibition through aberrant YAP activation.** (**A**) Representative images and quantification of foci formation in sgControl or sgMEF2D EpH4 cells. Scale bars = 200 µm. *** *p* < 0.001. (**B**) Immunofluorescence staining for Brdu in Brdu-labeled sgControl or sgMEF2D EpH4 cells. Scale bars = 25 µm. (**C**) Immunofluorescence staining for YAP in sgControl or sgMEF2D EpH4 cells. Scale bars = 25 µm. (**D**) Real-time qPCR analysis showed that MEF2D knockdown increased the mRNA expression of YAP/TAZ target genes Cyr61, Areg, and Ankrd1. ** *p* < 0.01. (**E**) Immunoblot detection of YAP knockdown efficiency by siRNA in sgMEF2D EpH4 cells. GAPDH was used as a loading control. Representative images and quantification of 3D acini formation of siYAP in sgMEF2D EpH4 cells. Scale bars = 100 µm.

Increased cell proliferation and tumor formation resulting from MEF2D knockout prompted us to investigate whether the loss of MEF2D function led to a loss of contact inhibition. We plated sgControl or sgMEF2D EpH4 cells in two-dimensional (2D) culture plates and allowed them to grow to confluence. The control cells stopped growing once they reached confluence (Figure 4A). In contrast, the sgMEF2D EpH4 cells continued to proliferate and formed foci (Figure 4A). Next, to verify that sgMEF2D maintained its cell proliferation capacity, we labeled the sgControl sgMEF2D EpH4 cells with bromodeoxyuridine (BrdU), a thymidine analog that incorporates into the DNA of dividing cells during the S phase of the cell cycle [27]. As expected, we found that the sgControl-EpH4 cells ceased the cell cycle under confluent cell conditions. However, BrdU was actively incorporated in the sgMEF2D EpH4 cells under the same growth conditions (Figure 4B).

The loss of contact inhibition is a hallmark of cancer cells, and the Hippo pathway has been implicated in regulating this process. The Hippo pathway is a kinase signaling cascade that controls the activation of transcription factors YAP/TAZ, which promote cell proliferation. Additionally, the Hippo pathway is regulated by various cellular and non-cellular mechanisms, including cell polarity, soluble factors, mechanical forces, and intercellular-junction-associated proteins E-cadherin and α-catenin. To better understand the role of MEF2D in these processes, we performed immunostaining and found that YAP was localized in the cytoplasm of the sgControl EpH4 cells. However, YAP was nuclear-localized in the sgMEF2D EpH4 cells under the high cell density condition (Figure 4C). Consistent with these findings, we detected increased mRNA expression levels of CYR61, AREG, and ANKRD1, which are downstream canonical transcriptional targets of YAP/TAZ (Figure 4D). Correspondingly, the knockdown of YAP expression by siRNA in the sgMEF2D EpH4 cells completely inhibited the loss of MEF2D-function-induced branching and invasive 3D structures (Figure 4E). Together, these findings suggest that the depletion of MEF2D leads to the loss of contact inhibition, promoting cell overgrowth and tumorigenesis through YAP activation.

### 2.5. Knockdown of MEF2D Altered the Transcriptome and Oncogenic Signaling Pathways in MECs

**Figure 5:**
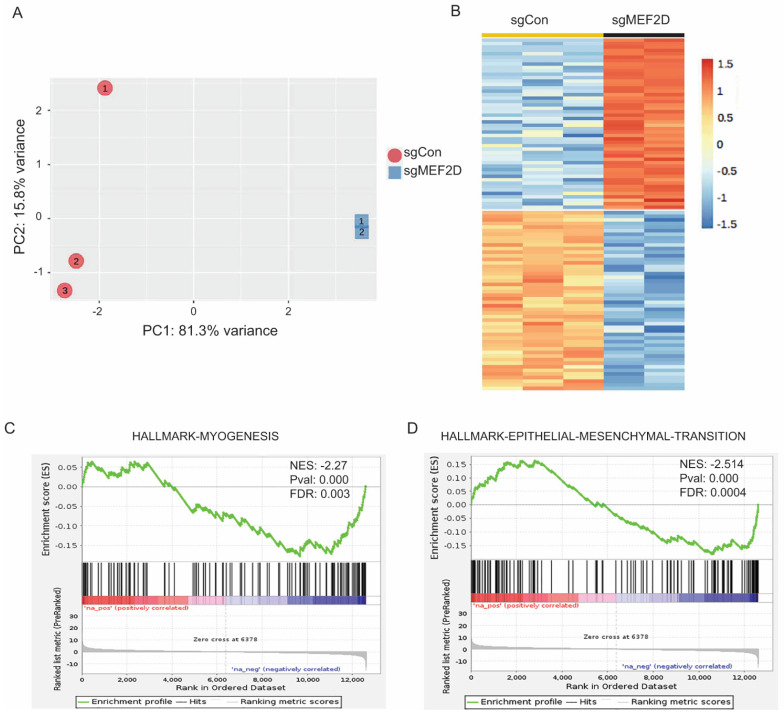
**RNA-seq analysis identified MEF2D-regulated gene expression and pathways.** (**A**) Principal component analysis (PCA) plot of the MEF2D-driven transcriptome in EpH4 cells. (**B**) Heatmap categorizing MEF2D up- or down-regulated gene expression. GSEA plots showing loss function of MEF2D reduced the myogenesis hallmark pathway (**C**); and reduced the EMT pathway (**D**).

To determine MEF2D-dependent gene expression programs, we harvested the RNA from the sgControl and sgMef2d EpH4 cells and performed an RNA-seq analysis. The principal component analysis (PCA) plot represents distinct transcription programs in response to MEF2D knockout, with a high reproducibility of each sample (Figure 5A). We identified 2097 significantly differentially expressed genes (*p* < 0.05) (Supplementary Table S1). Representative up-regulated and down-regulated gene expressions are shown in Figure 5B. To determine the MEF2D-regulated signaling pathways, we conducted a Hallmark pathways analysis using a gene set enrichment analysis (GSEA) (Supplementary Table S1). As expected, we found that the myogenesis pathway was significantly reduced in sgMef2d EpH4 cells, consistent with the role of the MEF2 transcription factor in regulating myogenic gene expression (Figure 5C; Supplementary Figure S3A,B). Furthermore, we confirmed the loss of MEF2D-induced EMT alterations (Figure 5D). Taken together, our RNA-seq analysis suggested that the loss of MEF2D contributes to tumorigenesis through the deregulation of several interconnected oncogenic signaling pathways.

### 2.6. Low Expression of MEF2D in Triple-Negative Breast Cancer Patients Was Correlated with Poor Breast Cancer Outcomes

**Figure 6:**
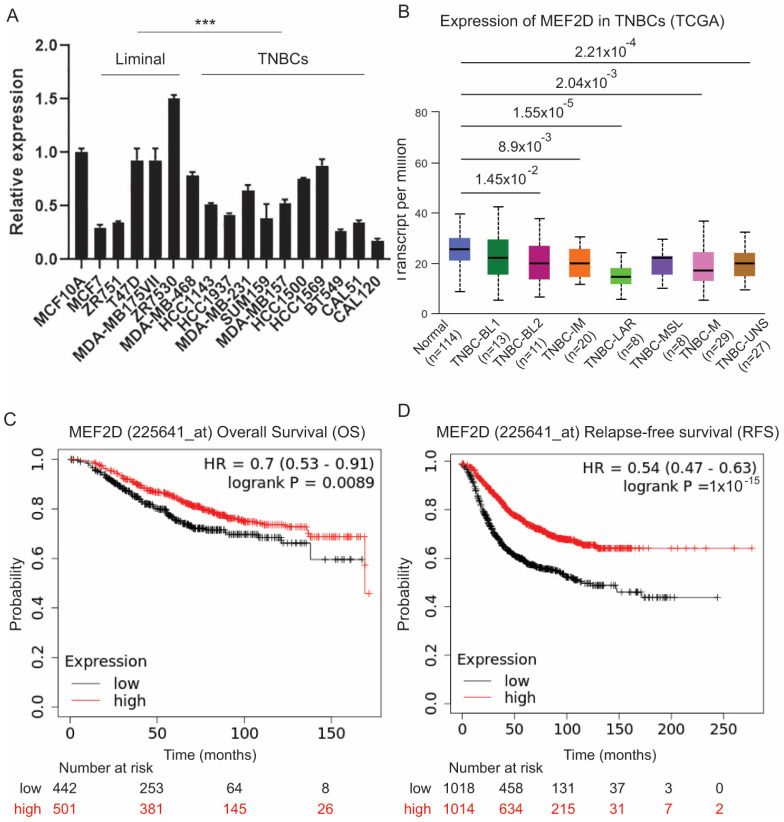
**Low MEF2D expression in TNBC and correlations with worse outcomes for breast cancer patients.** (**A**) MEF2D expression across a collection of different subtypes of breast cancer cell lines, including liminal and TNBC types. *** *p* < 0.001; one-way ANOVA analysis. (**B**) MEF2D expression was analyzed in TNBC subtypes using UALCAN. Correlations of MEF2D expression with overall survival (OS) (**C**) and relapse-free survival (**D**) in breast cancer patients obtained with Kaplan–Meier plots.

To confirm our findings in human breast cancer, we conducted a real-time qRT-PCR analysis on a set of well-characterized breast cancer cell lines. As shown in Figure 6A, the MEF2D expression was significantly lower in triple-negative breast cancer (TNBC) cells compared with normal (MCF10A) or estrogen-receptor-positive (ER+) breast cancer cells. We further analyzed the MEF2D mRNA expression levels in the TCGA breast cancer (BRCA) cohort using the UALCAN data analysis portal [28,29]. Consistently, we found that the expression of MEF2D was significantly lower in the TNBC samples compared with the normal breast tissue samples (Supplementary Figure S2B). Further analysis revealed that MEF2D expression was lower in several TNBC subtypes, including the TNBC basal-like 2 (BL2), TNBC immunomodulatory (IM), TNBC luminal androgen receptor (LAR), TNBC mesenchymal (M), and TNBC unstable (UNS) subtypes (Figure 6B). Finally, to determine whether MEF2D expression correlates with breast cancer patients’ outcomes, we analyzed the correlation between the MEF2D mRNA expression level and breast cancer patients’ survival using the Kaplan–Meier plotter [28]. As expected, we found that a low expression of MEF2D was correlated with a worse overall and relapse-free survival in breast cancer patients (Figure 6C,D).

## 3. Discussion

MEF2D is deregulated in different neoplasms and is associated with tumor progression and poor prognoses [3]. However, the direct role of MEF2D in breast cancer development and progression is poorly understood. In the present study, we showed that the perturbation of MEF2D leads to neoplastic transformation and mammary tumorigenesis through multiple potential mechanisms, including the activation of the AKT/ERK signaling pathway, induction of EMT, and loss of contact inhibition induced by YAP activation.

Many studies, including research conducted in our laboratory, have shown that EMT plays an important role in triggering tumor invasion and metastasis [30,31]. We found that the loss of MEF2D enhanced cell migration and EMT, as evidenced by the results of the cell migration and wound healing assays, and typical morphological changes. Consistent with these findings, the loss of MEF2D repressed the expression of epithelial-specific genes, such as E-cadherin and β-catenin, or induced the expression of mesenchymal-specific genes, and it decreased the expression levels of membrane-bound E-cadherin and β-catenin. It is interesting to note that, MEF2C, one of the members of the MEF2 family, was recently reported to be involved in the development of breast cancer brain metastases (BCBM) [32]. Together with our findings, this suggests that MEF2 family members may play crucial roles in breast cancer progression and metastasis.

It has long been recognized that the cell–cell adhesion molecule E-cadherin is an important tumor suppressor. The downregulation or loss of E-cadherin is a critical step in initiating the EMT, which enhances the invasive and metastatic abilities of breast cancer cells [33,34]. However, the overexpression of MEF2D has also been shown to induce the EMT in colorectal cancer, potentially through the transcriptional activation of ZEB1 [16]. This discrepancy between studies indicates that the cancer driver function of MEF2D is cell-type-specific and context-dependent.

Breast cancer involves the dysregulation of a variety of signaling pathways that play key roles in disease progression and the development of treatment resistance. For example, signaling pathways such as Wnt/β-catenin, NF-κB, Notch, Hedgehog, TGF-β, and Hippo play crucial roles in maintaining the self-renewal capacity of breast cancer stem cells. Furthermore, genetic alterations in pathways such as syndecan-1-mediated signaling, hepatocyte growth factor receptor signaling, and growth hormone signaling contribute to the predisposition to breast cancer. Similarly, the activation of the PI3K, Stat3, and Ras signaling pathways has been associated with different subtypes of breast cancer. A thorough understanding of these signaling pathways is essential for the development of effective treatments for breast cancer.

In this study, we explored whether MEF2D, a common upstream molecule, modulates known oncogenic signaling pathways associated with breast cancer. Specifically, we found that the loss of MEF2D increased the levels of p-ERK and p-AKT, indicating that MEF2D knockdown potentially activates the AKT and ERK pathways. Furthermore, we found nuclear-localized YAP in MEF2D-depleted EpH4 cells, which indicates that YAP was aberrantly regulated under the MEF2D knockdown condition.

The Hippo signaling pathway was recently identified as one of the mechanisms regulating the loss of contact inhibition [35,36]. YAP/TAZ are transcriptional coactivators and key effectors of the Hippo pathway. Nuclear-localized YAP/TAZ activates cell proliferation and anti-apoptosis gene expression [37]. In ovarian cancer, it has been reported that MEF2D expression is associated with cisplatin resistance [38]. We have previously shown that abnormal activation of YAP/TAZ confers chemotherapy resistance in breast cancer [39,40]. Further investigation is warranted to determine whether MEF2D plays an essential role in chemotherapy resistance in breast cancer through the activation of YAP/TAZ signaling.

Several molecular mechanisms involved in the Hippo pathway regulate the loss of contact inhibition. First, the activation of canonical upstream MST1/2-LATS1/2 kinases phosphorylates YAP/TAZ, leading to the cytoplasmic sequestration and protein degradation of YAP/TAZ [35]. Second, adherens junctions and the cadherin–catenin complex interact with YAP/TAZ, leading to the YAP/TAZ nuclear exclusion of YAP/TAZ [41,42]. Third, cell shape and mechanotransduction regulate Hippo–YAP signaling [43]. In the present study, we observed reduced protein levels and the decreased membrane localization of E-cadherin and β-catenin proteins in MEF2D knockdown cells, which may have resulted in nuclear YAP localization.

Signaling pathways are highly interconnected, forming complex networks that allow cells to respond to various internal and external stimuli. Notably, the AKT and ERK signaling pathways can contribute to the regulation and activation of YAP. For example, when activated, the PI3K/AKT/mTOR pathway can inhibit the Hippo pathway, resulting in the dephosphorylation and activation of YAP. AKT can phosphorylate and inhibit components of the Hippo pathway, such as LATS1/2 (large tumor suppressor kinase 1 and 2), preventing them from phosphorylating and inhibiting YAP. Consequently, YAP remains unphosphorylated, allowing it to translocate into the nucleus and induce gene expression that promotes cell proliferation and survival. Similarly, the ERK signaling pathway, which is part of the MAPK pathway, may also interact with the components of the Hippo pathway. Activated ERK can lead to the phosphorylation of specific proteins that regulate the Hippo pathway, thus influencing YAP activity. The direct mechanisms by which ERK influences YAP can be context-dependent and may involve multiple potential intermediates, warranting further investigation.

The identification of potential TSGs, such as MEF2D, provides the opportunity to develop drugs that target specific driver genes in breast cancer. Since oncogenes function through a gain of function (by being overly active or overly abundant), drugs have been designed to inhibit these activities. Conversely, TSGs contribute to cancer through loss of function. When tumor suppressor genes are mutated or deleted, their protective functions are lost or diminished. This lack of function does not present a direct “target” for traditional small-molecule drugs or antibodies because there is no excess activity to inhibit. Due to the complexity of restoring or compensating for lost tumor suppressor function, an alternative strategy is to target the pathways it normally regulates. The identification of therapeutic vulnerabilities based on deregulated signal transduction pathways has facilitated the development of highly effective targeted drugs. In this regard, we identified several oncogenic signaling pathways that can be targeted, warranting further investigation in breast cancer tumors with deregulated MEF2D.

In summary, our findings indicated that the genetic perturbation of MEF2D led to the transformation of MECs in vitro and the development of mammary tumors in vivo. Consistent with these findings, the expression of MEF2D mRNA was significantly lower in TNBCs compared with normal breast tissue in the TCGA cohort dataset. Furthermore, the low expression of MEF2D was correlated with a poor overall survival and recurrence-free survival in breast cancer patients. Hence, this study provides compelling evidence supporting the role of MEF2D as a promising target for the development of new therapeutic strategies in not only breast cancer, but also other types of cancer.

## 4. Materials and Methods

### 4.1. Cell Culture and Transfection

EpH4 and HC11 cells were purchased from the American Type Culture Collection (ATCC, Manassas, VA, USA), and 293FT cells were purchased from Thermo Fisher Scientific (Grand Island, NY, USA). The cells were grown in Dulbecco’s Modified Eagle Medium (DMEM) growth media with 10% fetal bovine serum (FBS) and 1% penicillin-streptomycin (10,000 U/mL) (Gibco; Grand Island, NY, USA) and incubated in 5% CO_2_ at 37 °C. Lentiviral particles expressing Cas9, sgControl, or sgMEF2D were generated in the 293FT cells by co-transfecting the Cas9-expressing vector or single-guide RNA (sgRNA) constructs with ecotropic packaging plasmids in accordance with standard protocols. The virus-containing medium was harvested 48 h after transfection. Cas9-containing virus particles were used to infect the EpH4 or HC11 cells, and stable clones were then selected after incubation in 5 ug/mL of blasticidin for five days. sgCon or sgMEF2D virus particles were used to infect the Cas9-expressing EpH4 or HC11 cells, and the stable clones were selected after incubation in 2 ug/mL of puromycin for five days; these cells were used for further functional analysis.

The sgRNA target sequences were as follows (Table 1):

siControl, siYAP#1 (hs.Ri.YAP1.13.1) and siYAP#2 (hs.Ri.YAP1.13.2) were purchased from Integrated DNA Technologies (IDT; San Diego, CA, USA). DharmaFECT™ 1 (Horizon Discovery, Lafayette, LA, USA) was used to transfect siRNAs into the cells according to the manufacturer’s instructions.

### 4.2. Immunoblot

A Western blot analysis was performed as described previously [44]. Briefly, cell lysates were harvested in a RIPA buffer (Boston Bio-Products, Milford, MA, USA) in the presence of protease and phosphatase inhibitors (Thermo Fisher Scientific, NY, USA), and the protein concentration was determined. Approximately 50 μg of protein lysate was loaded and separated by sodium dodecyl sulfate-polyacrylamide gel electrophoresis (SDS-PAGE), and then the product was transferred onto polyvinylidene fluoride (PVDF) membranes (EMD Millipore, Burlington, MA, USA). The transferred PVDF membranes were blocked by 5% milk at room temperature for 1 h and incubated overnight with a specific primary antibody at 4 °C. Next, the membranes were washed three times with tris-buffered saline with Tween (TBST) and incubated with horseradish peroxidase (HRP)-congregated anti-mouse or anti-rabbit secondary antibody (Bio-Rad, Hercules, CA, USA) for 1 h at room temperature. Proteins were detected using an ECL (enhanced chemiluminescence) Western blotting substrate (Thermo Fisher Scientific, NY, USA). The following antibodies were used in this study: MEF2D, E-cadherin, and β-catenin antibodies (BD Biosciences, Milpitas, CA, USA); vimentin, AKT, p-AKT, ERK, and p-ERK antibodies (Cell Signaling Technology, Danvers, MA, USA); GAPDH (UBPBio, Dallas, TX, USA); β-actin (Abcam, Waltham, MA, USA); and anti-bromodeoxyuridine (Brdu) (G3G4) antibodies (Developmental Studies Hybridoma Bank, Iowa City, IA, USA).

### 4.3. Cell Proliferation Assay

The cells were seeded in 96-well plates with 1500 cells per well and cultured for 1–7 days. Then, 10 μL of resazurin solution (Sigma, Saint Louis, MO, USA) was added to the medium of each well (final concentration = 44 µm) and incubated for 4 h at 37 °C. Absorbance was measured at 560 nm and 590 nm with a plate reader.

### 4.4. Soft agar Assay

A total of 2 mL of 0.5% agar was added to six-well plates as the base layer, and 2.0 × 10^4^ cells were seeded in 0.4% top agar (1.5 mL per plate). In addition, 1 mL of medium with 0.4% agar was added to the top of each plate every five days for two weeks. The colonies were stained with 0.02% iodonitrotetrazolium chloride solution (Sigma-Aldrich, Saint Louis, MO, USA), photographed, and counted.

### 4.5. Immunofluorescence Staining Assay

Cells grown on glass coverslips were fixed with 4% paraformaldehyde and permeabilized with 0.1% Triton X-100. Then, the cells were blocked with 2% bovine serum albumin (BSA) and incubated with anti-Brdu, anti-YAP, anti-E-cadherin, or anti-β-catenin antibodies overnight at 4 °C. After three washes with phosphate-buffered saline (PBS), the cells were incubated with Alexa Fluor 488-conjugated anti-mouse secondary antibody or Alexa Fluor 594-conjugated anti-rabbit secondary antibody (Thermo Fisher Scientific, NY, USA) for 1 h at room temperature. DAPI (4ʹ,6-diamidino-2-phenylindole) was used to stain the cell nuclei. Images were captured using a Leica fluorescent microscope (Leica Microsystems, Inc., New York, NY, USA).

### 4.6. Quantitative Reverse Transcription PCR (qRT-PCR)

The total RNA was extracted from the cells with TRIzol, and the cDNA was synthesized using a first-strand cDNA synthesis kit (GE Healthcare, Chicago, IL, USA) according to the manufacturer’s protocols. Real-time PCR reactions were performed using SYBR Green PCR master mix (Applied Biosystems, New York, NY, USA) on the ABI Prism 7900 HT system (Applied Biosystems). The experiments were repeated in triplicate, and the relative gene expression was calculated using the ΔΔCt method and standardized to the levels of GAPDH. The primer sequences (F, forward; R, reverse) were as follows (Table 2):

### 4.7. 3D Morphogenesis Assay

The cells were seeded in 8-well 3D culture chambers (Thermo Fisher Scientific, NY, USA) with ~5000 cells per well and cultured in a growth-factor-reduced Matrigel matrix (BD Biosciences). Images were acquired and analyzed on the eighth day of the cell culture. Spheres and stellate branch structures greater than 50 μm in diameter were counted.

### 4.8. Tumor Growth Assay

All animal experimental protocols were approved by the Institutional Animal Care and Use Committee of the Roswell Park Comprehensive Cancer Center in Buffalo, NY, USA. For the tumor growth assay, 1 × 10^6^ cells were resuspended in PBS 1:1 mixed with Matrigel (BD Biosciences) and injected into the 4th mammary fat pad of 6–8-week-old SCID mice (n = 6 mice/group). The tumor size was monitored every seven days using a dial caliper and calculated using the following formula: volume = width^2^ × length × 1/2.

### 4.9. RNA Extraction and Transcriptome Profiling

The total RNA of the EpH4-sgControl or EpH4-sgMef2d cells was extracted using Trizol Reagent (Life Technologies, Grand Island, NY, USA) according to the manufacturer’s protocol. The RNAs were further processed for next-generation sequencing at Roswell Park’s genomics core facility. RNA sequencing (RNA-seq) reads that passed the quality filter from Illumina RTA were first processed using FASTQC (v0.10.1) for sequencing the base quality control. Then, the sample reads were aligned to the mouse reference genome (GRCm38) and GENCODE (version 22) annotation database using STAR2 [45]. A second round of quality control (QC) using RSeQC [46] was applied to the mapped bam files to identify potential RNA-seq library preparation problems. Gene-level raw counts were obtained by using the gene set enrichment analysis (GSEA) [47] pre-rank tool and were used to run the analysis, i.e., the ranked gene data that pre-filtered the low count genes. A pathway analysis was run against MSigDB, a collection of annotated and curated gene set repositories offered by the developer of GSEA (Broad Institute of MIT and Harvard). This particular run used three databases of the version 2023.2 collection, namely, the Canonical Pathway (CP), Hallmark Pathway, and Transcription Factor Targets (TFT).

### 4.10. Clinical Data Analysis

An MEF2D gene expression analysis was performed on the Cancer Genome Atlas (TCGA) breast cancer cohort using the University of Alabama at Birmingham Cancer (UALCAN) Data Analysis Portal [28,29]. The breast cancer patients’ survival analyses were performed using the Kaplan–Meier plotter [48].

### 4.11. Statistical Analysis

SPSS version 16 for Windows was used for the statistical analysis. Each experiment was independently repeated at least three times. The results are presented as the mean ± standard error of the mean (SEM). Statistical significance was evaluated by using a Student’s *t*-test (two-tailed) or one-way analysis of variance (ANOVA) to compare the different groups of data. A *p*-value < 0.05 was considered as statistically significant.

## Figures and Tables

**Table 1:** **sgControl and sgMEF2D sequenses**.

	sgRNA Sequences
sgControl:	GCGAGGTATTCGGCTCCGCG
sgMEF2D-#1:	CATCAGTCCAAACTTCCGCT
sgMEF2D-#2:	TGCAGGTGACCTTCACCAAG

**Table 2:** **qPCR primer sequences.**

qPCR Primers	Forward	Reverse
Mouse		
Cyr61	CCA GTG TAC AGC AGC CTA AA	CTG GAG CAT CCT TAA GTA A
Areg	CCA TCA TCC TCG CAG CTA TT	CTT GTC GAA GCC TCC TTC TT
Ankrd1	GCCTACAAGAACTCTCGCATA	GTT GCT CTT CTG TTG GGA AAT G
Gapdh	AAC AGC AAC TCC CAC TCT TC	CCT GTT GCT GTA GCC GTA TT
Human		
MEF2D	CGAGAT CGC ACT CAT CAT CTT	TCG TGT GGC TCA TTG TAC TC
GAPDH	GTGAAGGTCGGAGTCAACGG	GAGGTCAATGAAGGGGTCATTG

## Data Availability

The raw RNA-seq data presented in the study were deposited in the Gene Expression Omnibus (GEO) repository (GSE267037). The original contributions presented in the study are included in the article/Supplementary Material, further inquiries can be directed to the corresponding author/s.

## Supplementary Materials

The supporting information can be downloaded at: https://www.mdpi.com/article/10.3390/ijms25105207/s1.
