# Supplementary material for: MEF2D Functions as a Tumor Suppressor in Breast Cancer
Source: Int J Mol Sci. 2024 May 10;25(10):5207. doi: 10.3390/ijms25105207 (PMC11121549; doi:10.3390/ijms25105207)
Supplement: Supplementary file 1 [file ijms-25-05207-s001.zip › Supplemental Figures and Legends.pdf]

Supplemental Figures and Figures Legends

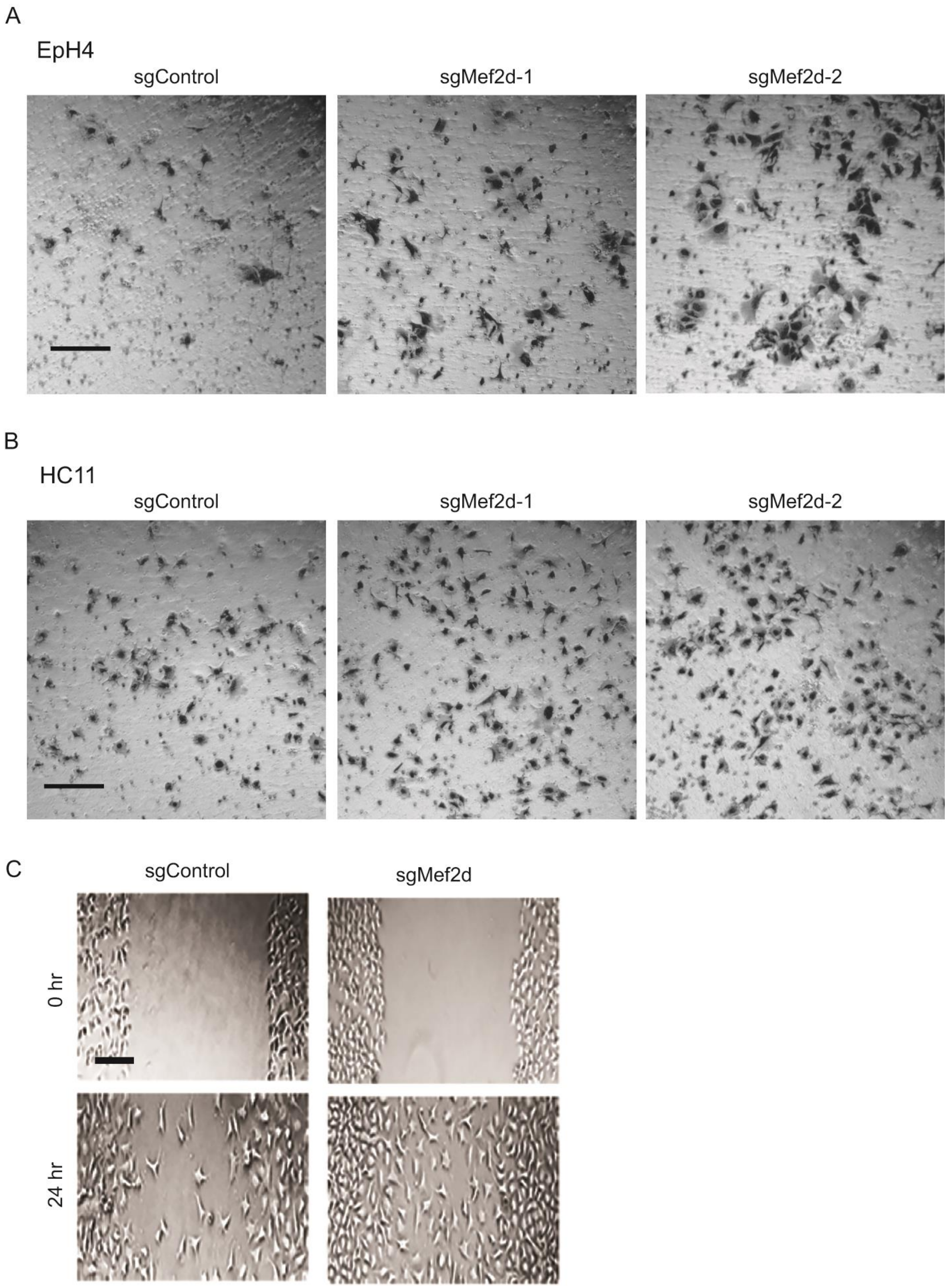

Figure S1. Knockdown of MEF2D increased cell migration in murine EpH4 and HC11 cells. A. Representative images of Boyden chamber cell migration assays with sgControl or sgMEF2D EpH4 cells. Scale bar=100μm **B.** Representative images of Boyden chamber cell migration assays with sgControl or sgMEF2D HC11 cells. Scale bar=100μm **C.** Representative images of wound healing assays demonstrated that loss of MEF2D increased EpH4 cell migration. Scale bar=100μm

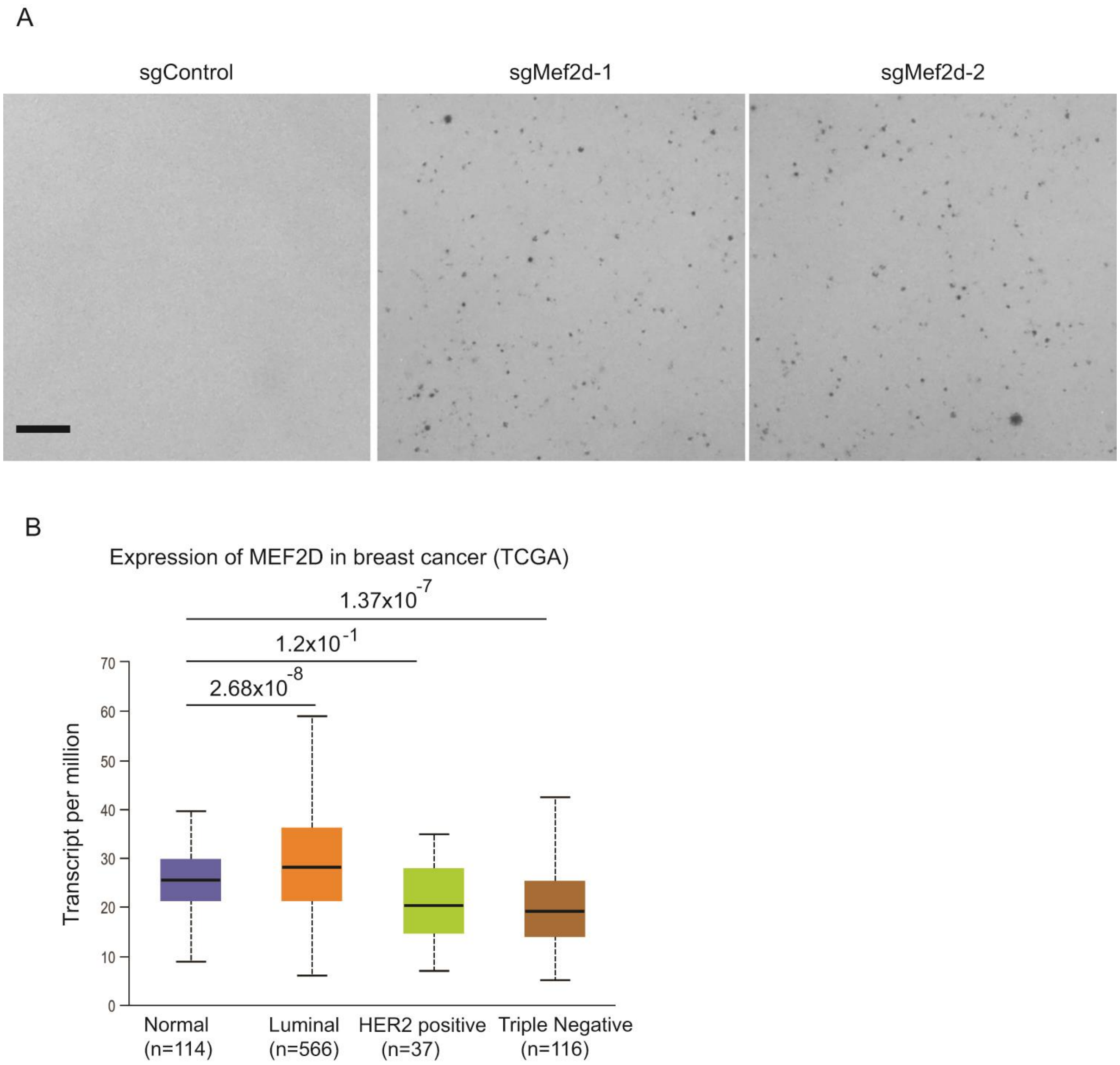

Figure S2. Knockdown of MEF2D increased colony formation in soft agar. A. Representative images of colony formation in soft agar for sgControl or sgMEF2D EpH4 cells. Scale bar=1mm **B.** MEF2D expression was analyzed in different subtypes of TCGA breast cancer patient samples in UALCAN.

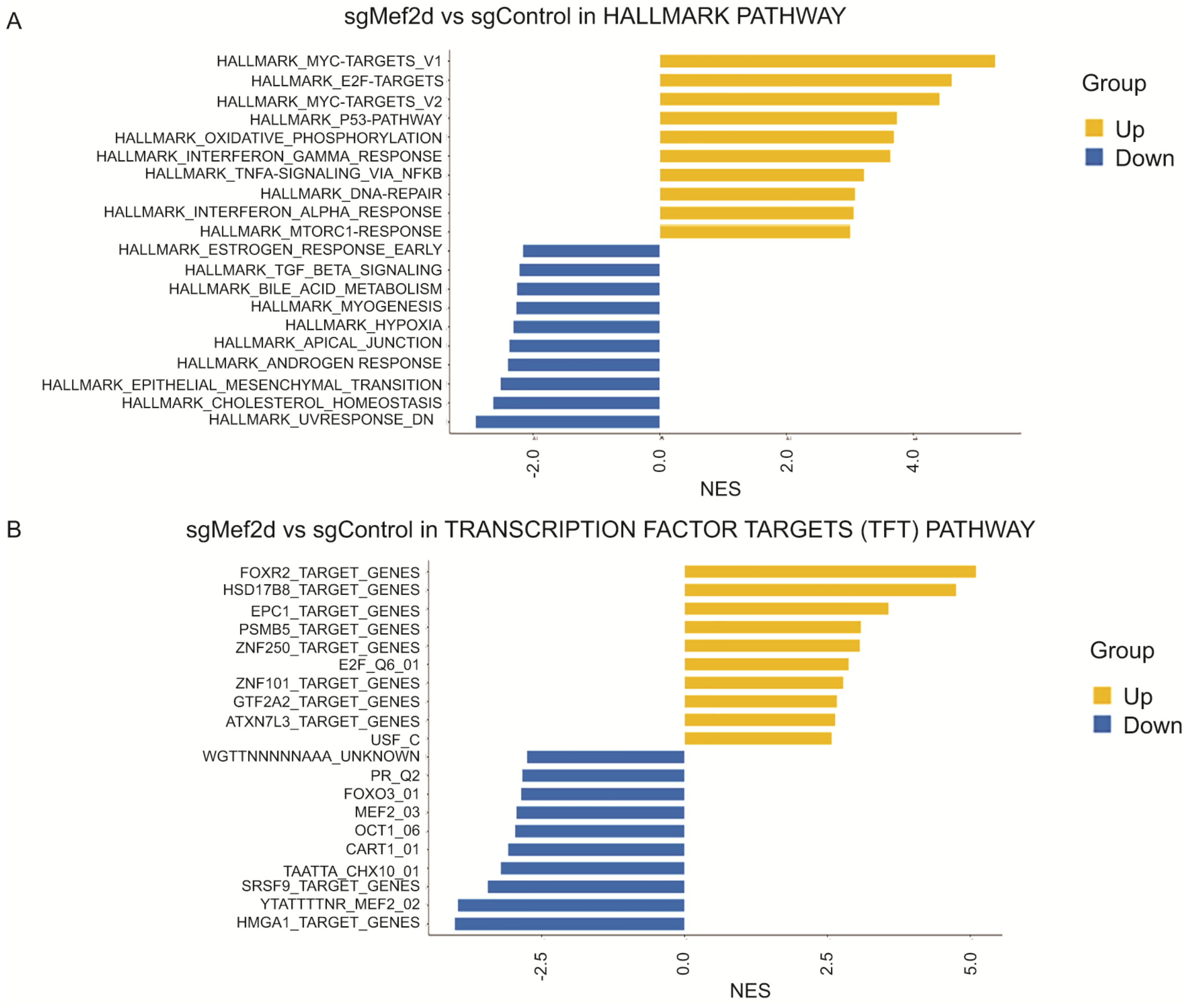

Figure S3. GSEA analysis identified MEF2D regulated pathways. A. sgMef2d- versus sgControl-regulated hallmark pathways. **B.** sgMef2d versus sgControl transcription factor target (TFT) pathways.
